# Novel biosurfactant-stabilized nanoemulsions integrating interfacial stabilization and antibacterial activity for safe surface disinfection^☆^

**DOI:** 10.1016/j.ultsonch.2026.107938

**Published:** 2026-06-28

**Authors:** Geum-Jae Jeong, Hyo-Jin Kim, Ye-Hyeon Jo, Se-Chang Kim, Dong-Joo Park, Kyung-Jin Cho, Na-Yeon Kim, Jung-Min Lee, Minji Kim, Won-Kyo Jung, Young-Mog Kim

**Affiliations:** a Department of Food Science and Technology, Pukyong National University, Busan 48513, the Republic of Korea; b Marine Integrated Biomedical Technology Center, The National Key Research Institutes in Universities, Pukyong National University, Busan 48513, the Republic of Korea; c Research Center for Marine Integrated Bionics Technology, Pukyong National University, Busan 48513, the Republic of Korea; d Interdisciplinary Program of Blue Food, Pukyong National University, Busan 48513, the Republic of Korea; e National Marine Biodiversity of Korea, Seochun 33662, the Republic of Korea; f Major of Biomedical Engineering, Division of Smart Healthcare, College of Information Technology and Convergence and New-senior Healthcare Innovation Center (BK21 Plus), Pukyong National University, Busan 48513, the Republic of Korea; g Korea Food Research Institute, Wanju 55365, the Republic of Korea

**Keywords:** Biosurfactant, Nanoemulsion, Natural disinfectant, Surface disinfection, Antibacterial activity

## Abstract

Chemical disinfectants are widely used for microbial control. However, concerns regarding their safety and environmental impact have driven the development of alternative antimicrobial systems based on natural components. Here, a biosurfactant (BS) derived from *Bacillus velezensis* GJ1 was used to construct a natural grade nanoemulsion (NE) system using thyme oil as the oil phase. Unlike conventional formulations in which surfactants mainly act as passive stabilizers, BS in this system contributed to both interfacial stabilization and antibacterial activity, enabling the development of a natural-grade NE system with combined physicochemical and antibacterial functionalities. The resulting NEs exhibited stable physicochemical properties and consistent droplet characteristics. Antibacterial evaluation demonstrated strong activity against *Listeria monocytogenes* and *Escherichia coli* O157, with comparable efficacy across different co-surfactant systems. This suggests that BS may contribute to the antibacterial performance of the NE systems. Microscopic analyses confirmed the membrane disruption and increased permeability of the bacterial cells. Under contact surface conditions, the BS-containing NEs exhibited measurable antibacterial activity against both *L. monocytogenes* and *E. coli* O157 under the tested conditions. NEs exhibited low cytotoxicity, minimal skin irritation, and negligible phytotoxicity at antibacterial concentrations. The findings suggest the potential applicability of BS-stabilized NEs as natural antimicrobial systems in which the surfactant contributes to both interfacial stabilization and antibacterial activity. These results may provide useful insights for the development of effective and safer disinfectant formulations based on naturally derived components.

## Graphical abstract

**Figure:**
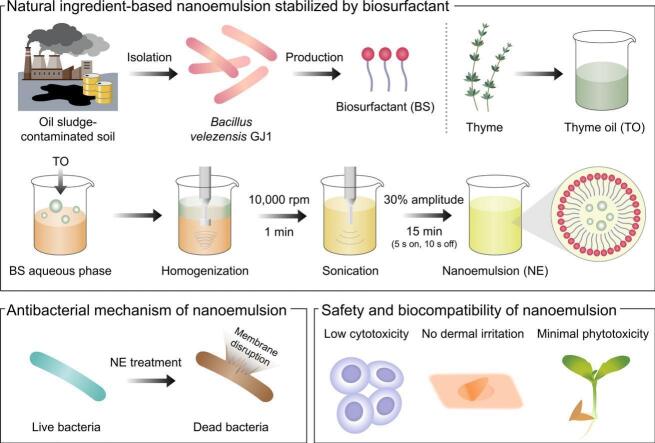

## 1 Introduction

Chemical disinfectants play a central role in controlling microbial contamination in healthcare- and food-related environments. Unlike antibiotics, which target specific pathways, disinfectants act on multiple cellular targets, particularly the cytoplasmic membrane, enabling rapid microbial inactivation [1]. However, their effectiveness is highly dependent on usage conditions such as concentration and exposure time [2]. Under suboptimal conditions, disinfectants may fail to achieve complete inactivation, and instead impose selective pressure on microbial populations [3]. These limitations highlight the need for alternative disinfection strategies that can ensure effective microbial control under practical conditions, while minimizing unintended safety and environmental impacts.

Nanoemulsion (NE) systems are attracting increasing attention as effective platforms for enhancing the delivery and antimicrobial performance of hydrophobic bioactive compounds. Owing to their small droplet size and large interfacial area, NEs can facilitate improved dispersion and interaction of active agents with microbial cells, enhancing antibacterial efficacy [4]. Essential oils have been widely incorporated into NE systems because of their inherent antimicrobial properties. The nanoscale structure of NEs enables more efficient contact between oil-phase compounds and bacterial membranes. This may enhance interactions with microbial membranes and promote increased permeability compared to bulk systems [4], [5]. Ultrasonication has been widely utilized as an effective emulsification technique for the preparation of NE systems because acoustic cavitation and shear-induced mechanical effects can facilitate droplet disruption and the formation of fine dispersions [6]. Compared with conventional mechanical homogenization methods, ultrasound-assisted emulsification can improve droplet size reduction and dispersion stability while operating under relatively mild processing conditions [7]. In addition, ultrasonication has been increasingly applied in essential oil-based NE systems to improve the dispersion and antimicrobial performance of hydrophobic bioactive compounds [8], [9], [10]. Nevertheless, most ultrasound-assisted NE studies have primarily focused on physicochemical stabilization and delivery efficiency, while the broader functional contribution of biosurfactants (BSs) within these systems remains relatively underexplored.

BSs are promising alternatives to synthetic surfactants due to their biodegradability, low toxicity, and environmental compatibility [11], [12]. In addition to their emulsifying capacities, certain BSs exhibit intrinsic antibacterial activities, including membrane disruption and increased cellular permeability [13], [14], [15]. Several recent studies have explored the use of BSs such as rhamnolipids and sophorolipids in antimicrobial NE systems [16], [17]. However, these studies mainly focused on physicochemical stabilization and antimicrobial delivery performance. Nevertheless, the integration of *Bacillus*-derived BSs as components contributing to both interfacial stabilization and antibacterial activity within a single NE system remains relatively underexplored.

In this context, we explored the potential application of BS as a multifunctional component contributing to both interfacial stabilization and antibacterial activity within NE systems. Unlike conventional NE formulations that rely on separate functional components, this design links interfacial properties with antibacterial performance. This strategy provides a framework for developing simplified, yet effective, antimicrobial systems composed entirely of naturally derived ingredients.

**Fig. 1:**
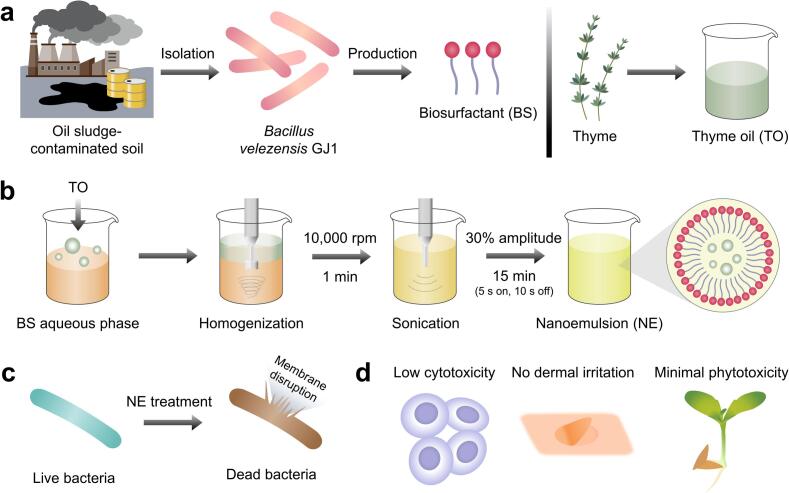
**Schematic illustration of the development and antibacterial application of BS-stabilized NEs. (a)** Use of the BS-producing strain *Bacillus velezensis* GJ1 and TO as key components for NE preparation. **(b)** Fabrication of BS-based NEs through homogenization and ultrasonication of the BS aqueous phase with TO. **(c)** Antibacterial mechanism of the NEs involving disruption of bacterial membrane integrity leading to cell death. **(d)** Safety profile of the formulated NEs, demonstrating low cytotoxicity, absence of dermal irritation, and minimal phytotoxicity.

In this study, BS produced by *Bacillus velezensis* GJ1 was used to construct a natural-grade NE system using thyme oil (TO) as the oil phase. The ability of BS to function as both a stabilizing agent and antibacterial component was investigated, along with its compatibility with other natural surfactants. The physicochemical properties, antibacterial activity, mechanism of action, and safety profiles of the resulting NE systems were evaluated. The antibacterial performance of the developed NE system was compared with that of sodium hypochlorite (200 ppm). This corresponds to the maximum concentration recommended by the U.S. Food and Drug Administration [18], using stainless steel as a representative contact surface. This approach enables the design of NE systems in which the surfactant contributes to antibacterial performance rather than solely being a stabilizing component. As illustrated in Fig. 1, the strategy encompasses BS production, NE fabrication, and an evaluation of antibacterial efficacy and safety, highlighting the potential of BS-based NEs as natural disinfectant formulations. This approach highlights the potential relationship between interfacial functionality and antibacterial performance in BS-based NE systems.

## 2 Materials and methods

### 2.1 Materials

Dulbecco’s modified Eagle’s medium (DMEM), fetal bovine serum (FBS), fluorescein diacetate (FDA), Nile Red, penicillin, propidium iodide (PI), streptomycin, and SYTO 9 were purchased from Thermo Fisher Scientific (USA). Hydrochloric acid, lecithin (LEC), saponin (SAP), sodium dodecyl sulfate (SDS), sodium hydroxide, and sodium hypochlorite were purchased from Sigma-Aldrich (USA). Lead citrate, osmium tetroxide, propylene oxide, a sodium cacodylate buffer, and uranyl acetate were purchased from Electron Microscopy Sciences (USA). Chloroform and methanol were purchased from Duksan Pure Chemicals (Republic of Korea). Formaldehyde and glutaraldehyde were purchased from Wako Pure Chemical (Japan). Tryptic soy agar (TSA) and tryptic soy broth (TSB) were purchased from BD Difco (USA). TO was purchased from a local market (Republic of Korea).

### 2.2 Microorganisms

The BS-producing strain, *B. velezensis* GJ1, was isolated from oil sludge-contaminated soil, and its identity was confirmed by both 16S rRNA gene sequencing (GenBank accession No. PV785483) [19] and whole-genome sequencing (GenBank accession No. JBVUOD000000000) [20]. Representative Gram-negative and Gram-positive bacteria, *Escherichia coli* O157 (15739) and *Listeria monocytogenes* (13064), respectively, were used to evaluate the antibacterial activity of the NEs. *E. coli* O157 was obtained from the National Culture Collection for Pathogens (Republic of Korea) and *L. monocytogenes* was obtained from the Korean Collection for Type Cultures (Republic of Korea). Glycerol stocks of all strains were maintained at −80 °C. Before the antibacterial assays, *E. coli* O157 and *L. monocytogenes* were streaked onto TSA and incubated to obtain single colonies. A single colony of each strain was inoculated in TSB and incubated at 37 °C with shaking at 150 rpm for 8 h. The resulting bacterial cultures were used as the inocula for subsequent experiments.

### 2.3 Production and partial purification of BSs

BS was produced by *B. velezensis* GJ1 and partially purified *via* acid precipitation and solvent extraction [21], as described in the materials and methods section of the supplementary material. The chemical structure of BS produced by *B. velezensis* GJ1 has been previously characterized using Fourier-transform infrared spectroscopy (FTIR), liquid chromatography-mass spectrometry, and nuclear magnetic resonance analyses, which identified surfactin C as the major component [19].

### 2.4 Preparation of NEs

NEs were prepared using TO and BS derived from *B. velezensis* GJ1 to construct a natural antibacterial system. The chemical composition of TO was analyzed using gas chromatography-mass spectrometry (GC-MS), as described in the supplementary materials. For NE preparation, a BS-containing aqueous solution was first prepared as the surfactant phase and combined with Milli-Q water to form a continuous phase. TO was then added as the oil phase and the mixture was subjected to high-speed homogenization at 10,000 rpm for 1 min to obtain a coarse emulsion. The coarse emulsion was further processed using a probe-type sonicator (Vibra-Cell, SONICS, USA; 20 kHz) equipped with a 3 mm probe tip at 30% amplitude in pulse mode, that is, 5 s on, 10 s off, for a total sonication time of 15 min. Sonication was performed in an ice bath to minimize the temperature increase, and the sample temperature was maintained below 25 °C throughout the ultrasonication process (Fig. S1). Calorimetric estimation indicated that the actual acoustic power under the applied sonication conditions was approximately 2.1 W (Table S1). The corresponding total energy input and energy density were estimated to be approximately 633 J and 63.3 J/mL, respectively (Table S1). Pulse-mode ultrasonication and moderate amplitude conditions were applied to promote efficient emulsification while limiting excessive thermal accumulation during processing [22]. A total volume of 10 mL coarse emulsion was subjected to ultrasonication for each preparation batch. Ultrasonication was performed using a 50 mL conical tube with an approximate probe immersion depth of 1 cm. To examine the influence of the formulation parameters, NEs were prepared by varying the BS:TO ratio (1:1, 2:1, 4:1, 8:1, and 16:1, *V*/*V*) and BS concentration (1, 5, 10, 15, 20, 25, and 30 mg/mL). The BS:TO ratio and BS concentration were evaluated separately to distinguish the effects of relative composition and absolute BS concentration on NE formation. The resulting NEs were characterized by measuring their hydrodynamic diameters, polydispersity index (PDI), and zeta potentials using a Litesizer 500 (Anton Paar, Austria). Based on these characteristics, a representative formulation was selected for subsequent characterization and antibacterial evaluation. The selected formulation (BS = 16:1, *V*/*V*; BS concentration = 20 mg/mL) contained approximately 2.5% (*V*/*V*) TO in the final NE system. For the compatibility assessment, additional NEs were prepared by incorporating natural surfactants, including LEC and SAP, combined with BS under identical preparation conditions. For the mixed-surfactant formulations, BS and the additional natural surfactant (LEC or SAP) were each incorporated at 10 mg/mL in the aqueous phase prior to emulsification, corresponding to approximately 0.4 wt% each (0.8 wt% total surfactant content) in the final formulation. To further elucidate the interfacial role of BS in NE formation, the interfacial tension (IFT) between TO and aqueous solutions containing BS and natural surfactants, either individually or in combination, was measured. The detailed measurement procedures are described in the supplementary materials.

### 2.5 Physicochemical and morphological characterization

#### 2.5.1 Cryo-TEM

The morphology of NEs was examined by cryogenic-transmission electron microscopy (Cryo-TEM). A small aliquot of the sample was deposited onto a glow-discharged copper grid (GloQube Plus; Quorum Technologies, UK; 15 mA for 60 s). Excess liquid was blotted using a Vitrobot system (Thermo Fisher Scientific, USA) under controlled conditions (chamber temperature: 1 °C; blot time: 10 s; blot force: 1; wait time: 15 s). This was followed by rapid vitrification in liquid ethane. The vitrified grids were transferred to a TEM (Talos L120C; Thermo Fisher Scientific, USA) equipped with a cryoholder (Elsa 698; Gatan, USA). Images were acquired at an accelerating voltage of 120 kV.

#### 2.5.2 CLSM

The microstructures of the NEs were examined using confocal laser scanning microscopy (CLSM; LSM 900, Zeiss, Germany) [23]. To visualize the oil phase, the NE samples were stained with Nile red at a final concentration of 20 μg/mL and incubated in the dark for 20 min. Following staining, a sample aliquot was deposited on a glass slide and overlaid with a coverslip before CLSM observation. Nile Red was excited at 530 nm for fluorescence imaging.

#### 2.5.3 FTIR

FTIR spectroscopy was used to analyze the functional groups and possible interactions between TO, BS, and the prepared NEs. Spectra were recorded in attenuated total reflectance mode using an FT-4100 spectrometer (Jasco, Japan) over a wavenumber range of 4,000–650 cm^−1^. Samples were freeze-dried prior to FTIR analysis.

### 2.6 Stability evaluation

#### 2.6.1 Thermal stability

The thermal stability of the NEs was evaluated by subjecting the samples to heat treatment at 60, 80, 100, 120, and 140 °C for 60 min. After heating, the samples were cooled to 25 °C and visually inspected for any signs of phase separation and macroscopic instability. In addition, the hydrodynamic diameter, PDI, and zeta potential were measured after treatment to evaluate changes in physicochemical stability.

#### 2.6.2 pH stability

The pH stability of the NEs was assessed by adjusting the pH of the samples to 3, 5, 7, 9, and 11 using 0.1 M hydrochloric acid or sodium hydroxide solutions. After equilibration at 25 °C, the samples were visually examined for phase separation and instability. The hydrodynamic diameter, PDI, and zeta potential were also measured following pH adjustment.

#### 2.6.3 Thermodynamic stability (heating-cooling cycles)

Thermodynamic stability was further evaluated through heating-cooling cycles. The NEs were alternately stored at 45 °C for 48 h and at 4 °C for 48 h, constituting one cycle. This process was repeated for five cycles. After each cycle, the samples were visually inspected for any evidence of phase separation or instability. Changes in hydrodynamic diameter, PDI, and zeta potential were additionally monitored throughout the cycling process.

### 2.7 *In vitro* antibacterial activity and mechanism evaluation

#### 2.7.1 Agar well diffusion

The antibacterial efficacy of NEs was evaluated using an agar well diffusion assay. Suspensions of *L. monocytogenes* and *E. coli* O157 were prepared at approximately 6 log colony forming units (CFU)/mL and evenly spread onto TSA. Under aseptic conditions, wells were formed in the agar, and each well was loaded with 100 μL of sample. The plates were incubated at 37 °C for 24 h after which the diameters of the inhibition zones were measured. Sterile distilled water was used as a negative control.

#### 2.7.2 MIC and MBC

The MIC and MBC of NEs were determined against *L. monocytogenes* and *E. coli* O157 using the broth microdilution method. Bacterial cultures were adjusted to approximately 6 log CFU/mL in TSB and the samples were serially diluted two-fold in 96-well microplates containing TSB. An equal volume of bacterial suspension was added to each well, and wells containing untreated bacteria were growth controls. Plates were incubated at 37 °C for 24 h, and bacterial growth was assessed by measuring optical density at 600 nm. The MIC was defined as the lowest concentration resulting in a ≥ 90% reduction in growth compared with the control. For MBC determination, aliquots from wells showing no visible growth were plated onto TSA and incubated at 37 °C for 24 h. The MBC was defined as the lowest concentration at which no colonies were observed.

#### 2.7.3 FE-SEM

Morphological changes in bacterial cells after treatment with NEs were examined using field-emission scanning electron microscopy (FE-SEM) [24]. Bacterial suspensions of *L. monocytogenes* and *E. coli* O157 (approximately 6 log CFU/mL) were exposed to the NEs at 1/2 × MIC and incubated at 37 °C for 24 h under static conditions. Untreated bacterial suspensions were used as the controls. After incubation, the bacterial cells were collected and fixed overnight at 4 °C in a solution containing 2.5% glutaraldehyde and 2% paraformaldehyde. Samples were washed twice with phosphate-buffered saline (PBS) and subsequently dehydrated using a graded series of ethanol solutions of 30%, 50%, 70%, 80%, 95%, and 100%. The dehydrated samples were dried, sputter-coated with platinum, and observed using a FE-SEM (JSM-IT800SHL, JEOL, Japan) at an accelerating voltage of 3 kV.

#### 2.7.4 CLSM

Membrane damage in *L. monocytogenes* and *E. coli* O157 following treatment with NEs was evaluated by CLSM. Bacterial cultures were harvested by centrifugation (13,700 × *g*, 15 min), washed twice with PBS, and resuspended in PBS. The bacterial suspensions were exposed to the NEs at the MBC level and maintained at 37 °C for 4 h. Subsequently, the cells were harvested, rinsed with PBS, and stained with SYTO 9 (5 μM) and PI (2 μg/mL) in 200 μL of staining solution for 15 min under dark conditions. Following the removal of excess dye by centrifugation (6,100 × *g*, 4 min) and subsequent washing with PBS, a portion of the suspension was mounted on a glass slide, overlaid with a coverslip, and analyzed using CLSM. Untreated bacterial suspensions in PBS were used as the controls.

#### 2.7.5 Bio-TEM

Changes in the membrane structure and cellular integrity of *L. monocytogenes* and *E. coli* O157 following NE treatment were analyzed using Bio-TEM. Bacterial samples were prepared as described in Section 2.7.4. Detailed procedures for sample pretreatment and imaging are provided in the supplementary materials.

### 2.8 Antibacterial performance on contact surfaces

The antibacterial performances of contact surfaces were evaluated using a surface inoculation model. Before use, stainless-steel coupons (2 × 2 × 0.1 cm) were disinfected with 70% ethanol for 15 min, rinsed with sterile distilled water, and air-dried. Suspensions of *L. monocytogenes* or *E. coli* O157 (1 × 10^6^ CFU/mL) were prepared. A defined volume was inoculated onto the surface of each coupon to achieve an initial contamination level of approximately 5 log CFU/cm^2^. The inoculated coupons were held under static conditions at 37 °C for 4 h to allow initial bacterial attachment to the stainless-steel surfaces prior to treatment. Following attachment, the coupons were treated with the test solutions, including NE formulations and sodium hypochlorite (200 ppm) as a reference disinfectant, at 25 °C for 5 min. After treatment, attached bacterial cells were recovered by vigorous vortexing in PBS for 2 min, followed by serial dilution, spread plating on TSA, and incubation at 37 °C for 24 h. Bacterial counts are expressed as log CFU/cm^2^.

### 2.9 Safety and biocompatibility assessment

#### 2.9.1 *In vitro* cytotoxicity

The cytotoxicity of the NEs was evaluated in human dermal fibroblasts (HDF) and RAW 264.7 macrophages. Cells were cultured in DMEM supplemented with 10% FBS and 1% penicillin-streptomycin at 37 °C in a humidified incubator with 5% CO_2_. Cells were seeded in 12-well plates at a density of 3 × 10^4^ cells/mL and allowed to attach for 24 h. Test solutions were prepared at the highest MBC determined against the tested pathogens. After 24 h of exposure, cell viability was measured using the Cell Counting Kit-8 assay (CCK-8; Dojindo, Japan). The culture medium was replaced with 100 μL of CCK-8 working solution and incubated for 2 h, and absorbance was measured at 450 nm. Cell viability was calculated relative to that of untreated controls. For visualization, a live/dead staining assay was performed using FDA (5 μg/mL) and PI (20 μg/mL) for 5 min. The stained cells were then rinsed with PBS and observed using CLSM.

#### 2.9.2 *In vivo* skin irritation test

The dermal irritation potential of the NEs was evaluated using a rat model. All the procedures were conducted in accordance with institutional guidelines and approved by the Institutional Animal Care and Use Committee of Pukyong National University (Approval No. PKNUIACUC-2025-02). Before treatment, the dorsal hair was shaved to expose intact skin. Sterile filter papers (1.0 × 1.0 cm) soaked with undiluted BS-NE, BS-LEC-NE, or BS-SAP-NE were placed on the dorsal region (approximately 1.5 × 1.5 cm). Filter paper soaked in 10% (w/v) SDS and sterile distilled water served as the reference irritant and negative control, respectively. After 24 h of exposure, the paper was removed and the skin was visually inspected for erythema and edema.

#### 2.9.3 Phytotoxicity evaluation

The phytotoxicity of NEs was evaluated using *Brassica rapa* seeds. Test solutions were prepared at the highest MBC determined against the tested pathogens and diluted in sterile distilled water. Seeds were surface sterilized with 95% ethanol and 2% sodium hypochlorite and rinsed with sterile distilled water. Ten sterilized seeds were placed in sterile Petri dishes containing Whatman No. 1 filter paper. This was followed by the addition of 5 mL of test solution to each dish. Seeds treated with sterile distilled water were the controls. The dishes were incubated at 25 °C for five days, after which the number of germinated seeds was recorded.

### 2.10 Statistical analysis

Statistical analyses were performed using GraphPad Prism (version 11.0.0). Group comparisons were performed using one-way analysis of variance (ANOVA), followed by Tukey’s or Dunnett’s post-hoc tests, as appropriate for the experimental design. Statistical significance was defined as *p < 0.05, **p < 0.01, ***p < 0.001, and ****p < 0.0001, where ns denotes no statistically significant difference.

## 3 Results and discussion

### 3.1 Fabrication and characterization of BS-stabilized NEs

**Fig. 2:**
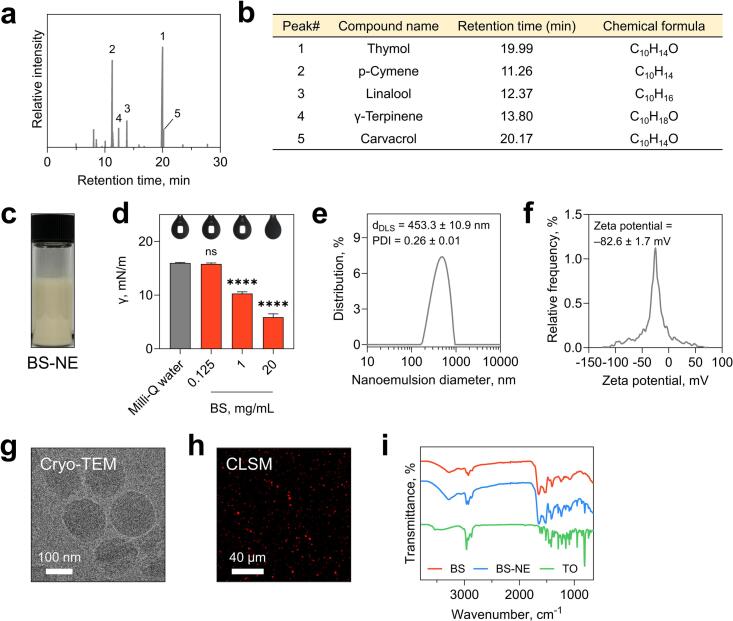
**Formation and physicochemical properties of BS-stabilized NEs. (a)** GC-MS chromatogram of TO. **(b)** Major chemical constituents of TO identified by GC-MS. **(c)** Visual appearance of the BS-NE formulation. **(d)** IFT between TO and aqueous BS solutions at different concentrations (*n* = 5). **(e)** Hydrodynamic diameter and PDI of the BS-NE determined by DLS. **(f)** Zeta potential of the BS-NE. **(g)** Cryo-TEM image showing the morphology of the NE droplets. **(h)** CLSM image of BS-NE stained with Nile red to visualize the oil phase. **(i)** FTIR spectra of TO, BS, and BS-NE indicating molecular interactions in the NE system. ****p < 0.0001 indicates a significant difference compared with Milli-Q water, while ns denotes no statistically significant difference.

TO was used as the oil phase to construct a natural-grade NE system based on BSs. The chemical composition of TO was first examined by GC-MS to identify its major constituents. As shown in Fig. 2a and b, TO contained several terpene- and phenolic-type compounds, with thymol, p-cymene, linalool, γ-terpinene, and carvacrol as the five major constituents. These compounds are widely recognized as the key bioactive constituents of TO and are known to contribute to its antimicrobial properties [25].

To examine the influence of the formulation parameters on NE formation, the BS-to-TO ratio and BS concentration were varied while maintaining identical preparation conditions. As shown in Fig. S2a and b, increasing the proportion of BS gradually decreased the hydrodynamic diameter of the emulsions. This is consistent with previous studies reporting that surfactants reduce the droplet size by lowering the IFT and inhibiting coalescence [26]. A similar tendency was observed when the BS concentration increased. The droplet size progressively decreased and approached a relatively stable range at higher concentrations (Fig. S3a and b). These observations suggest that the greater availability of BS at the interface contributes to improved dispersion of TO droplets during emulsification, possibly by suppressing droplet coalescence [26]. Based on these observations, a BS-to-TO ratio of 16:1 and BS concentration of 20 mg/mL were selected for subsequent characterization. The selected NE formulation (BS:TO = 16:1, *V*/*V*; BS concentration = 20 mg/mL) contained approximately 2.5% (*V*/*V*) TO in the final emulsion system. Ultrasonication is known to facilitate droplet disruption through acoustic cavitation and shear-induced mechanical effects during emulsification [27]. Under the applied sonication conditions, these effects likely promoted the formation of smaller dispersed droplets and improved dispersion stability by enhancing droplet breakup and limiting recoalescence. The selected formulation also maintained comparable hydrodynamic diameter, PDI, and zeta potential values after 24 h of storage, suggesting no apparent short-term physicochemical destabilization (Fig. S4a, b, and c).

Under these conditions, the BS-stabilized NE exhibited a homogeneous milky appearance without visible phase separation (Fig. 2c), suggesting the formation of a stable oil-in-water dispersion. The IFT between TO and the aqueous BS solutions was measured to further examine the interfacial behavior of BS during NE formation. As shown in Fig. 2d, the IFT decreased with increasing BS concentration, indicating the adsorption of BS molecules at the oil-water interface [28]. This interfacial activity may facilitate droplet breakup during homogenization and ultrasonication, contributing to the formation of dispersed NE droplets [29]. Under the applied ultrasonication conditions, acoustic cavitation and shear-induced mechanical effects may have contributed to the formation of smaller dispersed droplets during emulsification [6]. In addition, the presence of BS at the oil-water interface may have helped maintain droplet dispersion by reducing recoalescence following ultrasonication. The physicochemical properties of BS-NE were further analyzed using dynamic light scattering (DLS). As shown in Fig. 2e, the BS-NE exhibited an average hydrodynamic diameter of approximately 453 nm with a PDI of 0.26, indicating a moderately uniform droplet size distribution. NE displayed a highly negative zeta potential (−82.6 mV) (Fig. 2f). This suggests that electrostatic repulsion may contribute to the maintenance of dispersion stability. The morphology and microstructure of the NE droplets were examined using cryo-TEM and CLSM. Cryo-TEM images show spherical droplet-like structures dispersed in the aqueous phase (Fig. 2g), supporting the presence of dispersed oil droplets in the BS-stabilized system. Although the hydrodynamic diameter measured by DLS was approximately 453 nm, cryo-TEM observations revealed the presence of smaller dispersed droplets within the system. CLSM images obtained using Nile red staining to visualize the oil phase showed numerous fluorescent droplets distributed throughout the continuous phase (Fig. 2h), consistent with an oil-in-water-type dispersion [23]. These observations suggest that the BS promotes the dispersion of TO droplets *via* interfacial adsorption, resulting in a visually homogeneous and colloidally dispersed emulsion system. The FTIR spectra were analyzed to investigate the intermolecular interactions involved in the formation of the BS-stabilized NE (Fig. 2i). The FTIR spectrum of BS exhibited a broad absorption band in the 3,300–3,200 cm^−1^ region, which was associated with O–H and amide-related stretching vibrations linked to hydrogen bonding [30]. A characteristic band observed at approximately 2,926 cm^−1^ corresponded to aliphatic C–H stretching vibrations [19], while the absorption band near 1,645 cm^−1^ was associated with carbonyl stretching vibrations and/or bound water bending, reflecting the hydrophilic functional groups present in the BS structure [21]. The FTIR spectrum of TO displayed characteristic absorption bands representative of its essential oil composition. A broad absorption band in the 3,600–3,200 cm^−1^ region was associated with O–H stretching vibrations related to phenolic hydroxyl groups [31]. In addition, bands in the 2,960–2,870 cm^−1^ region corresponded to aliphatic C–H stretching vibrations arising from terpene-related constituents in TO [32]. After NE formation, the FTIR spectrum of BS-NE exhibited characteristic absorption bands corresponding to both BS and TO, suggesting the incorporation of TO into the BS-stabilized system. Notably, the hydroxyl stretching region of BS-NE showed slight broadening and a shift toward lower wavenumbers compared to that of TO, which may indicate the involvement of hydrogen bond-related intermolecular interactions after emulsification [23]. No new absorption bands or disappearance of characteristic peaks were observed in the BS-NE spectrum, indicating that NE formation occurred without chemical modification or covalent bond formation. Overall, these results suggest that the formation of BS-NE was associated with non-covalent intermolecular interactions, which may contribute to the stabilization and physical incorporation of TO within the BS-stabilized system while preserving the chemical integrity of the oil phase. These observations also suggest that the applied ultrasonication conditions primarily promoted physical emulsification and intermolecular interactions within the NE system without inducing detectable chemical modification of the formulation components.

### 3.2 Compatibility of BS-stabilized NEs with natural surfactants

**Fig. 3:**
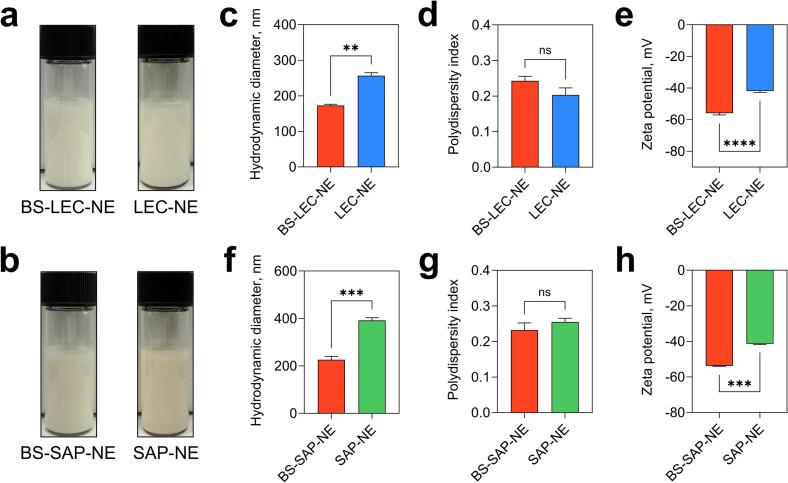
**Formation and droplet properties of BS-stabilized NEs derived from *Bacillus velezensis* GJ1 combined with natural surfactants. (a)** Visual appearance of BS-LEC-NE and LEC-NE. **(b)** Visual appearance of BS-SAP-NE and SAP-NE. **(c)** Hydrodynamic diameter, **(d)** PDI, and **(e)** zeta potential of BS-LEC-NE and LEC-NE (*n* = 3). **(f)** Hydrodynamic diameter, **(g)** PDI, and **(h)** zeta potential of BS-SAP-NE and SAP-NE (*n* = 3). **p < 0.01, ***p < 0.001, and ****p < 0.0001; ns indicates no significant difference.

To further evaluate the applicability of the BS-stabilized NE system, its compatibility with other natural surfactants was investigated using LEC and SAP as representative amphiphilic compounds. This approach has enabled the development of NE systems composed of natural components. Mixed surfactant systems are often used to improve emulsion stability and performance by modulating interfacial properties [33]. Therefore, in this study, the effects of combining BS with LEC or SAP on NE formation and droplet characteristics were examined. For the mixed surfactant systems, BS and the additional natural surfactants (LEC or SAP) were each incorporated at a final concentration of 10 mg/mL. As shown in Fig. 3a and b, both LEC-NE and SAP-NE had a turbid appearance. The corresponding BS-containing systems showed a comparable appearance, and no apparent phase separation was observed immediately after emulsification under the tested conditions. These observations suggest that the incorporation of BS does not adversely affect the emulsification behavior of natural surfactants. The droplet characteristics of the NEs were further analyzed using DLS. In the case of LEC-based systems, the incorporation of BS resulted in a decrease in the hydrodynamic diameter compared to LEC-NEs (Fig. 3c). Meanwhile, no noticeable difference was observed in PDI (Fig. 3d). A more pronounced effect was observed in the SAP-based systems, where BS-SAP-NE exhibited a smaller droplet size than SAP-NE (Fig. 3f). In contrast, the PDI values remained within a similar range. This suggests that the droplet size distribution was not significantly affected by the addition of BS (Fig. 3g). The zeta potential of the NEs became more negative upon the incorporation of BS in both systems, particularly in the SAP-based formulation (Fig. 3e and h). This change in surface charge may be associated with the adsorption of BS molecules at the droplet interface, which could increase electrostatic repulsion, helping maintain dispersion stability.

To further elucidate the interfacial behaviors of the mixed surfactant systems, IFT measurements were performed. In the LEC system, the IFT of the BS-LEC formulation was comparable to that of LEC alone, suggesting a limited reduction in the equilibrium IFT. Strong synergistic effects were not evident under the tested conditions (Fig. S5a). In contrast, the SAP-based system exhibited a reduction in the IFT upon the incorporation of BS. This suggested increased interfacial activity in the presence of BS (Fig. S5b). This reduction in the IFT may facilitate droplet disruption during emulsification, leading to the formation of smaller droplets. The smaller droplet sizes observed in the mixed surfactant formulations compared with the BS-only NE may therefore be attributed to the combined effects of interfacial tension reduction and improved interfacial organization in the presence of the coexisting natural surfactants. The anionic nature of BS may also contribute to increased electrostatic repulsion between droplets, whereas its interaction with SAP may reflect a more favorable mixed interfacial organization, as suggested by previous studies on mixed surfactant systems [34]. These results suggest that BS can function as a co-surfactant in natural surfactant-based NE systems and that its effectiveness depends on the type of coexisting surfactant. This effect appeared to be more pronounced in the SAP-containing system, which exhibited reduced IFT following BS incorporation, whereas the effect of BS incorporation in the LEC-based system was less pronounced, consistent with the relatively limited change observed in the IFT behavior.

### 3.3 Stability of BS-stabilized NE systems

**Fig. 4:**
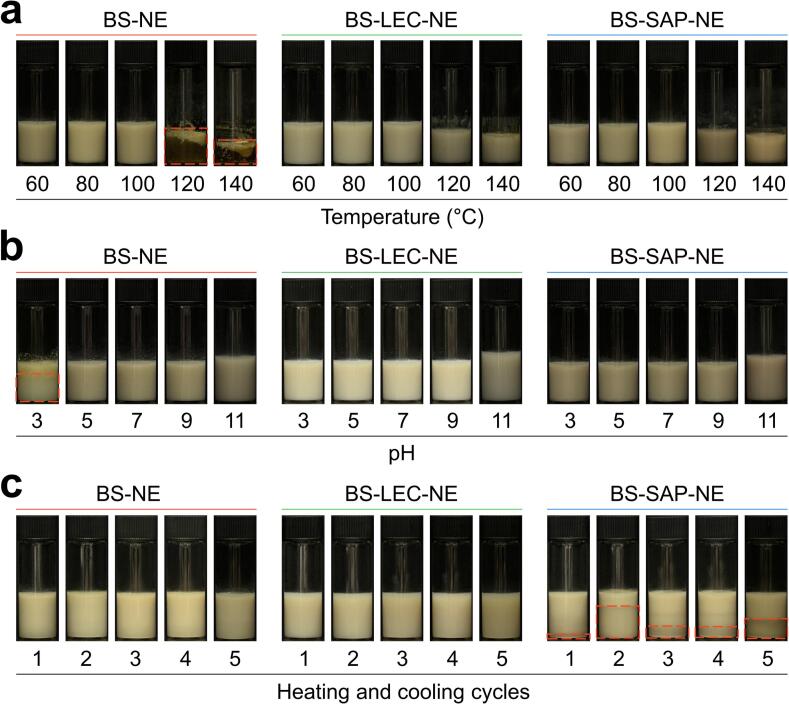
**Visual stability and phase behavior of BS-stabilized NE systems under various environmental conditions. (a)** Visual appearance of the NEs after thermal treatment at various temperatures. **(b)** pH stability of the NEs evaluated under different pH conditions. **(c)** Heating-cooling stability of the NEs subjected to repeated heating-cooling cycles. The red dashed boxes highlight conditions showing noticeable changes in stability.

The stabilities of the BS-stabilized NE systems under various environmental conditions were evaluated to assess their potential applicability. The thermal, pH, and thermodynamic stabilities were examined by monitoring the macroscopic phase behavior under different stress conditions, together with changes in hydrodynamic diameter, PDI, and zeta potential. As shown in Fig. 4a, the BS-NE remained visually stable without noticeable phase separation up to 100 °C. Meanwhile, phase separation was observed at higher temperatures of 120 and 140 °C. Consistent with these observations, gradual increases in droplet size were observed with increasing temperature prior to phase separation (Table 1). This instability at elevated temperatures may be associated with increased molecular motion, possible changes in the interfacial film integrity, and/or alterations in the oil phase under these conditions, which could facilitate droplet coalescence. In contrast, both the BS-LEC-NE and BS-SAP-NE systems showed no apparent phase separation over the tested temperature range. Although increases in droplet size were observed at elevated temperatures, the systems remained dispersed under the tested conditions (Table 1). The pH stability of the NE system is shown in Fig. 4b. BS-NE underwent phase separation under highly acidic conditions (pH 3), whereas it remained stable across the other tested pH conditions. This behavior may be associated with changes in the ionization state of the BS under acidic conditions, which could influence the surface charge and interdroplet interactions [35]. Both the BS-LEC-NE and BS-SAP-NE systems remained visually stable across the tested pH range. The corresponding physicochemical measurements also remained within comparable ranges under most tested pH conditions (Table 1). This suggests that the incorporation of additional natural surfactants may contribute to improved resistance to phase separation under acidic conditions. The thermodynamic stability was further evaluated through repeated heating-cooling cycles (Fig. 4c). The BS-NE and BS-LEC-NE systems remained stable over multiple cycles with no evident phase separation, although gradual changes in droplet size were observed during repeated cycling (Table 1). In contrast, the BS-SAP-NE system exhibited phase separation after repeated cycles, indicating reduced resistance to thermodynamic stress. This reduced stability may be associated with differences in the interfacial organization and/or dynamic rearrangement of the mixed surfactant system under repeated stress, which could influence the properties and integrity of the interfacial film [36]. These results suggest that the BS-based NE systems remain macroscopically stable under several tested environmental conditions, although their tolerance depends on the formulation composition and stress type. While BS alone provided sufficient stability under most conditions, its combination with LEC showed comparable stability. Meanwhile, the SAP-based system appeared to be more susceptible to thermodynamic stress under the tested conditions.

**Table 1:** Physicochemical characteristics of BS-stabilized NEs under various stability conditions.

**Condition**	**Level**	**BS-NE**			**BS-LEC-NE**			**BS-SAP-NE**		
		**Size (nm)**	**PDI**	**Zeta potential (mV)**	**Size (nm)**	**PDI**	**Zeta potential (mV)**	**Size (nm)**	**PDI**	**Zeta potential (mV)**
Thermal stability	60	476.43 ± 14.91^a^	0.29 ± 0.00^a^	−73.43 ± 0.29^b^	220.90 ± 7.23^c^	0.28 ± 0.01^a^	−40.97 ± 1.33^a^	278.23 ± 11.57^d^	0.25 ± 0.00^a^	−54.97 ± 5.23^ab^
	80	494.60 ± 37.92^a^	0.26 ± 0.02^a^	−69.93 ± 1.56^a^	271.93 ± 26.51^bc^	0.29 ± 0.01^a^	−40.40 ± 0.20^a^	408.33 ± 23.93^c^	0.27 ± 0.03^a^	−54.77 ± 3.30^ab^
	100	510.13 ± 19.88^a^	0.25 ± 0.03^a^	−68.40 ± 0.46^a^	295.43 ± 17.75^b^	0.28 ± 0.02^a^	−42.57 ± 0.25^a^	560.73 ± 20.43^b^	0.29 ± 0.02^a^	−57.23 ± 2.16^b^
	120	ND	ND	ND	456.47 ± 35.81^a^	0.28 ± 0.01^a^	−42.00 ± 1.15^a^	721.47 ± 11.77^a^	0.30 ± 0.03^a^	−47.67 ± 2.21^a^
	140	ND	ND	ND	487.33 ± 20.48^a^	0.28 ± 0.01^a^	−42.00 ± 1.15^a^	733.47 ± 20.60^a^	0.27 ± 0.01^a^	−48.00 ± 1.15^a^
pH stability	3	ND	ND	ND	190.88 ± 0.11^a^	0.23 ± 0.02^a^	−29.73 ± 0.29^a^	379.53 ± 9.67^a^	0.24 ± 0.01^a^	−34.83 ± 0.64^a^
	5	456.63 ± 31.38^a^	0.29 ± 0.01^a^	−77.87 ± 0.85^a^	164.12 ± 9.43^b^	0.22 ± 0.01^a^	−36.60 ± 2.78^b^	243.63 ± 11.18^b^	0.24 ± 0.02^a^	−44.57 ± 0.31^b^
	7	453.27 ± 10.86^a^	0.26 ± 0.01^a^	−82.57 ± 1.69^c^	172.98 ± 3.23^b^	0.24 ± 0.01^a^	−39.90 ± 0.17^bc^	225.93 ± 14.21^bc^	0.23 ± 0.02^a^	−53.77 ± 0.21^c^
	9	428.37 ± 17.27^ab^	0.28 ± 0.01^a^	−73.80 ± 0.10^b^	163.67 ± 5.33^bc^	0.23 ± 0.01^a^	−41.23 ± 0.38^c^	205.76 ± 15.38^c^	0.27 ± 0.02^a^	−73.83 ± 1.55^d^
	11	382.20 ± 6.32^b^	0.27 ± 0.01^a^	−75.77 ± 0.15^ab^	150.37 ± 1.28^c^	0.22 ± 0.01^a^	−55.97 ± 1.04^d^	164.18 ± 3.74^d^	0.27 ± 0.00^a^	−78.77 ± 1.29^e^
Thermodynamic stability (heating-cooling cycles)	1	584.47 ± 11.51^a^	0.26 ± 0.03^a^	−56.50 ± 2.85^a^	198.50 ± 3.47^a^	0.28 ± 0.01^a^	−41.10 ± 0.35^b^	ND	ND	ND
	2	596.63 ± 54.26^a^	0.29 ± 0.01^a^	−52.10 ± 0.60^a^	202.77 ± 1.54^a^	0.27 ± 0.01^a^	−41.70 ± 0.62^b^	ND	ND	ND
	3	586.13 ± 50.07^a^	0.27 ± 0.01^a^	−50.50 ± 5.52^a^	212.38 ± 15.47^a^	0.28 ± 0.02^a^	−39.50 ± 1.50^ab^	ND	ND	ND
	4	602.20 ± 59.03^a^	0.27 ± 0.01^a^	−50.63 ± 2.12^a^	215.33 ± 17.31^a^	0.28 ± 0.01^a^	−39.20 ± 2.23^ab^	ND	ND	ND
	5	666.63 ± 61.50^a^	0.30 ± 0.02^a^	−50.50 ± 1.23^a^	246.23 ± 15.18^a^	0.27 ± 0.01^a^	−35.63 ± 2.01^a^	ND	ND	ND

Values are presented as mean ± SD (*n* = 3). Different lowercase letters within the same sample group indicate significant differences among stability conditions (*p* < 0.05). ND, not determined due to phase separation.

### 3.4 *In vitro* antibacterial activity and mechanistic insights of BS-stabilized NEs

**Fig. 5:**
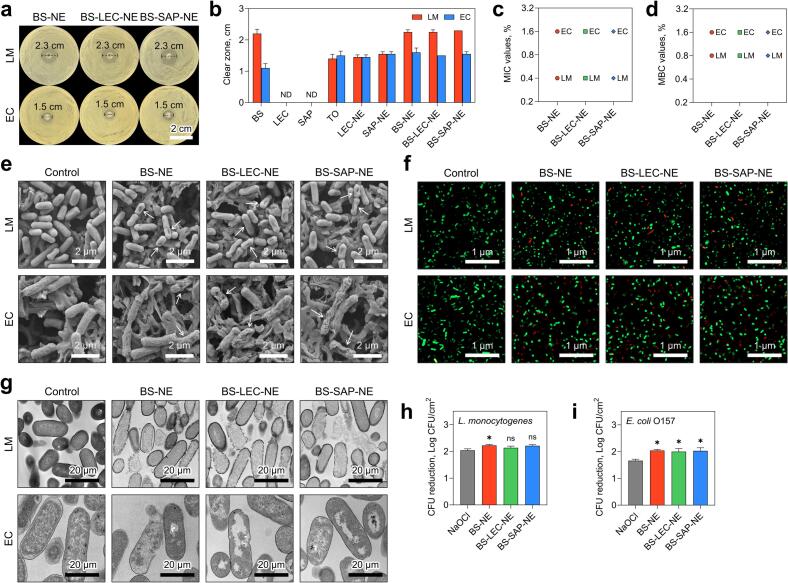
**Antibacterial performance and membrane-disruption mechanism of BS-stabilized NEs *in vitro* and contact surfaces. (a)** Agar diffusion assay showing inhibition zones against *Listeria monocytogenes* (LM) and *Escherichia coli* O157 (EC) treated with individual components and NE formulations. **(b)** Diameter of inhibition zones for each treatment against LM and EC (*n* = 3). **(c)** MIC and **(d)** MBC values of the NE formulations against LM and EC. **(e)** FE-SEM images showing morphological changes in LM and EC after treatment with NE formulations. Arrows indicate damaged or disrupted cell structures. **(f)** CLSM images of LM and EC stained with SYTO 9 (green, live cells) and PI (red, dead cells) after treatment with the NE formulations. **(g)** Bio-TEM images showing ultrastructural alterations of LM and EC cells following treatment with NE formulations. Log reduction of **(h)** LM and **(i)** EC on stainless steel surfaces after treatment with NaOCl (200 ppm) and NE formulations at their MBC concentrations (*n* = 3). *p < 0.05 indicates a significant difference compared with NaOCl, while ns denotes no statistically significant difference. ND denotes no detectable zone of inhibition.

To develop a natural-grade NE system as a potential alternative to conventional chemical disinfectants, the antibacterial activities of BS-stabilized NEs were evaluated against *L. monocytogenes* and *E. coli* O157. The system is composed exclusively of naturally derived components with the aim of providing an effective and safe surface disinfection strategy. As shown in Fig. 5a, b, and S6, BS alone exhibited pronounced antibacterial activity against *L. monocytogenes*, whereas TO alone showed moderate antibacterial activity against both strains. This observation is consistent with our previous findings on the strong antibacterial activity of BSs derived from *B. velezensis* GJ1, particularly against *L. monocytogenes* [19]. The NE formulations showed enhanced inhibition compared to natural surfactant-based systems without BS, suggesting that BS may contribute to the antibacterial performance of the NE systems. BS-containing NEs (BS-NE, BS-LEC-NE, and BS-SAP-NE) consistently produced strong inhibition zones against *L. monocytogenes*. Further assessments using the MIC and MBC assays supported these observations. As shown in Fig. 5c and d, the MIC and MBC values of the BS-containing NE formulations were comparable across different systems. This suggests that the antibacterial efficacy was largely preserved regardless of the type of co-surfactant incorporated. A similar trend was observed for both *L. monocytogenes* and *E. coli*.

This interpretation was further supported by microscopic analysis. FE-SEM images showed that all BS-based NE formulations induced similar morphological alterations in bacterial cells, including surface deformation and structural disruption, compared to the untreated controls (Fig. 5e). CLSM observations showed increased PI uptake in the treated cells, indicating compromised membrane integrity (Fig. 5f). These effects were consistently observed across all BS-containing formulations, with no distinct differences between them. Bio-TEM analysis provided additional insight into the mode of action (Fig. 5g). In *L. monocytogenes*, treatment with the NE formulations resulted in severe disruption of cellular structures, including deformation of the cell envelope and irregular intracellular organization. In contrast, *E. coli* cells generally retained their morphology while exhibiting reduced intracellular electron density, suggesting the leakage of cytoplasmic components without complete structural collapse. These differences may reflect differences in the membrane robustness or structural responses to membrane-active agents. These observations indicated that NE systems act primarily by increasing membrane permeability and disrupting cellular integrity. Similar mechanisms have been reported in previous studies on NE systems, where membrane disruption was considered a major mode of antibacterial action [17], [37], [38].

These results suggest that the antibacterial activity of BS-stabilized NEs may be associated with the presence of BS in combination with the intrinsic antibacterial activity of TO. The comparable antibacterial performance observed across the different BS-containing formulations indicates that the incorporation of additional natural surfactants did not substantially alter the antibacterial efficacy under the tested conditions. In this study, LEC and SAP alone did not exhibit detectable antibacterial activity (Fig. S6), suggesting that these surfactants primarily contributed to the physicochemical stabilization of the NE systems rather than directly contributing to antibacterial effects. Overall, the present findings support the potential application of BS-containing natural NE systems as functionally integrated antimicrobial formulations combining interfacial stabilization and antibacterial activity. These findings highlight the potential utility of BSs not only as stabilizing agents but also as functionally active components in natural antimicrobial NE systems.

### 3.5 Antibacterial efficacy of BS-stabilized NE systems on contact surfaces

The antibacterial performance of BS-stabilized NE systems on contact surfaces was evaluated using stainless steel as a representative material. For comparison, 200 ppm sodium hypochlorite, which corresponds to the maximum concentration recommended by the U.S. Food and Drug Administration for contact surfaces, was used as the reference [18]. As shown in Fig. 5h and i, all NE formulations exhibited consistent antibacterial activity against both *L. monocytogenes* and *E. coli* O157. For *L. monocytogenes*, the NE formulations achieved slightly higher log reductions of approximately 2.2 log CFU/cm^2^, than sodium hypochlorite at approximately 2.0 log CFU/cm^2^. For *E. coli*, the NE-treated groups showed higher log reduction values of approximately 2.0 log CFU/cm^2^ than those treated with sodium hypochlorite at approximately 1.7 log CFU/cm^2^. The differences among the NE formulations were minimal, indicating that the incorporation of additional natural surfactants did not influence the antibacterial performance under contact conditions. Although the antibacterial reduction levels differed slightly depending on the bacterial strain, all BS-containing NE formulations exhibited measurable antibacterial activity on stainless steel surfaces under the tested conditions. The observed antibacterial reduction levels were similar to those obtained with 200 ppm sodium hypochlorite within the experimental conditions evaluated in this study. These findings suggest the potential applicability of BS-stabilized NE systems as naturally derived antimicrobial surface treatment formulations.

The BS-stabilized NE systems exhibited measurable antibacterial activity on stainless steel surfaces under the tested conditions. These findings suggest the potential applicability of naturally derived BS-based NE formulations as antimicrobial surface treatment systems. In addition, the present results may provide useful insights into the development of natural antimicrobial formulations utilizing BS-based interfacial systems.

### 3.6 Safety of BS-stabilized NE systems

**Fig. 6:**
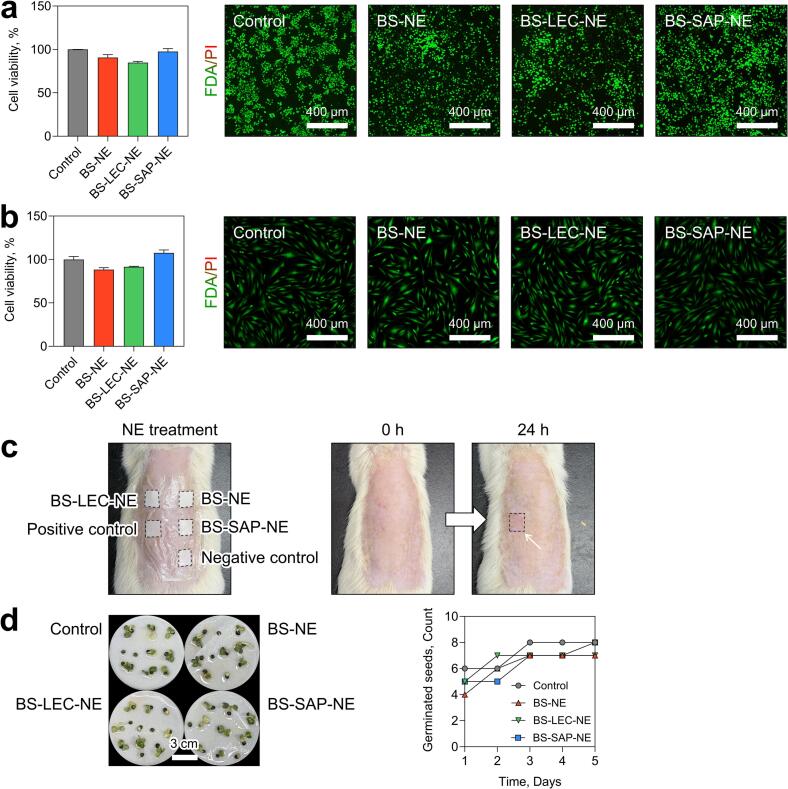
**Safety evaluation of BS-stabilized NE systems.** Live/dead staining images of **(a)** RAW 264.7 and **(b)** HDF cells treated with the NEs, visualized using FDA/PI staining, with the corresponding cell viability quantified in the bar graphs (*n* = 3). **(c)***In vivo* skin irritation assessment following topical application of NEs on dorsal skin, with representative images at 0 h and 24 h after treatment. **(d)** Seed germination assay evaluating the phytotoxicity of NE systems, including representative images of germinated seeds and quantitative analysis of germination over time (*n* = 10).

The balance between the antibacterial performance and biocompatibility is a key consideration in the development of NE-based disinfectant systems. The safety of BS-stabilized NE systems was evaluated to assess their suitability as natural-grade disinfectant formulations, considering both human and environmental aspects. Cytotoxicity was assessed at the MBC of the formulations (Fig. 6a and b). Cell viability remained above 70% for all NE systems, suggesting relatively low cytotoxicity. According to the ISO 10993-5 guidelines, materials with cell viability below 70% are considered cytotoxic [39]. Therefore, BS-stabilized NEs may be regarded as non-cytotoxic under the tested conditions. The antibacterial concentrations required for effective disinfection did not induce substantial toxicity in mammalian cells. To further examine potential membrane damage in mammalian cells, FDA/PI staining was performed. The results showed minimal PI uptake in the treated cells. This suggested that the NE formulations did not cause significant membrane disruption under the tested conditions. This observation contrasts with the membrane damage observed in bacterial cells, indicating a possible degree of selectivity toward microbial membranes. The skin irritation was evaluated using undiluted samples (Fig. 6c). No visible signs of erythema or edema were observed in the NE-treated group, whereas the positive control group showed clear irritation. These results suggest that BS-stabilized NE systems are unlikely to induce noticeable skin irritation and may be suitable for topical application. This observation is consistent with the limited membrane disruption observed in mammalian cells, suggesting a degree of selectivity toward microbial membranes. The environmental safety was assessed using phytotoxicity tests (Fig. 6d). Seed germination rates in the NE-treated groups were comparable to those in the untreated control, suggesting limited phytotoxic effects under the tested conditions. Considering that residual antimicrobial agents may enter the food chain and pose potential risks to human health [21], the low phytotoxicity observed in this study suggests that the developed NE systems showed limited phytotoxic effects under the tested conditions. These results indicate that BS-stabilized NE systems exhibit effective antibacterial activity while maintaining low cytotoxicity, minimal skin irritation, and limited environmental toxicity. These findings highlight the potential of the developed systems as naturally derived antimicrobial formulations with favorable biocompatibility profiles.

## 4 Conclusion

In this study, a natural-grade NE system stabilized by BS derived from *B. velezensis* GJ1 was successfully developed and evaluated for its physicochemical characteristics and antibacterial activity. The BS contributed to interfacial stabilization and antibacterial activity within the NE system. The NE structure enhanced the dispersion and interfacial accessibility of the BS, facilitating its interaction with bacterial membranes. The minimal variation in antibacterial efficacy across different co-surfactant systems highlights the potential of flexible formulation designs without compromising performance. The developed NEs exhibited measurable antibacterial activity on contact surfaces while maintaining low cytotoxicity, minimal skin irritation, and negligible phytotoxicity. This combination of efficacy and safety addresses a key limitation of conventional disinfectants, which often involves trade-offs between antibacterial activity and biocompatibility. This study highlights the potential application of BS not only as an interfacial stabilizer but also as a functionally active component in natural antimicrobial NE systems. The developed BS-stabilized NEs demonstrated measurable antibacterial activity while maintaining favorable safety profiles, suggesting their potential utility as naturally derived antimicrobial surface treatment formulations.

## CRediT authorship contribution statement

**Geum-Jae Jeong:** Writing – review & editing, Writing – original draft, Visualization, Validation, Methodology, Funding acquisition, Formal analysis, Data curation, Conceptualization. **Hyo-Jin Kim:** Validation, Methodology, Formal analysis, Data curation. **Ye-Hyeon Jo:** Validation, Methodology, Formal analysis, Data curation. **Se-Chang Kim:** Validation, Methodology, Formal analysis. **Dong-Joo Park:** Validation, Methodology, Formal analysis. **Kyung-Jin Cho:** Methodology, Formal analysis. **Na-Yeon Kim:** Methodology, Formal analysis. **Jung-Min Lee:** Methodology, Formal analysis. **Minji Kim:** Methodology, Formal analysis. **Won-Kyo Jung:** Writing – review & editing, Validation, Methodology, Funding acquisition. **Young-Mog Kim:** Writing – review & editing, Writing – original draft, Validation, Supervision, Methodology, Funding acquisition, Data curation.

## Declaration of competing interest

The authors declare that they have no known competing financial interests or personal relationships that could have appeared to influence the work reported in this paper.

## Appendix A Supplementary data

Supplementary Data 1 Supplementary material includes supplementary methods, additional tables, and figures that support the experimental procedures and results presented in the main manuscript.
