# Supplementary material for: Novel biosurfactant-stabilized nanoemulsions integrating interfacial stabilization and antibacterial activity for safe surface disinfection
Source: Ultrason Sonochem. 2026 Jun 28;131:107938. doi: 10.1016/j.ultsonch.2026.107938 (PMC13330536; doi:10.1016/j.ultsonch.2026.107938)
Supplement: Supplementary Data 1 — Supplementary material includes supplementary methods, additional tables, and figures that support the experimental procedures and results presented in the main manuscript. [file mmc1.docx]

**Supplementary materials for**

**Novel biosurfactant-stabilized nanoemulsions: integrating interfacial stabilization and antibacterial activity for safe surface disinfection**

Jeong *et al*.

Corresponding author: Young-Mog Kim

This file includes:

Materials and methods

Table S1

Fig. S1 to S6

**1. Supplementary materials and methods**

**1.1. Production and partial purification of BSs**

*Bacillus velezensis* GJ1 was cultured in TSB at 37 °C with shaking at 150 rpm for 120 h to produce BSs. The culture broth was centrifuged at 27,000 ×*g* for 20 min at 4 °C to remove bacterial cells, and the resulting supernatant was filtered through a 0.2 μm membrane to obtain a cell-free filtrate. The filtrate was acidified to pH 2.0 using 6 M hydrochloric acid and maintained at 4 °C overnight to induce precipitation of BS components. The precipitate was recovered by centrifugation under the same conditions and resuspended in deionized water. The pH was adjusted to 7.0 with 1 M sodium hydroxide prior to solvent extraction using a chloroform-methanol mixture (2:1, *V*/*V*), with the final chloroform:methanol:water ratio adjusted to 8:4:3 (*V*/*V*/*V*). After overnight extraction, the organic phase was collected and the solvent was removed under reduced pressure using a rotary evaporator. The resulting crude BS was freeze-dried and stored at −20 °C until further use.

**1.2. Chemical composition analysis of TO by GC-MS**

The chemical composition of TO was analyzed using a gas chromatograph-mass spectrometer (GCMS-QP2020 NX, Shimadzu, Japan) equipped with a DB-5MS capillary column (5% phenyl, 95% dimethylpolysiloxane; 30 m × 0.25 mm i.d. × 0.25 μm film thickness). Helium was used as the carrier gas at a constant flow rate of 1.0 mL/min. The injection was performed in split mode (200:1) with the injector temperature maintained at 250 °C. The oven temperature program was set as follows: an initial temperature of 50 °C held for 4 min, increased to 240 °C at a rate of 5 °C/min, followed by a ramp to 300 °C at 10 °C/min with a final hold of 7 min. The mass spectrometer was operated in electron ionization mode at 70 eV. Mass spectra were acquired in full scan mode over a mass range of m/z 40–550. The ion source and interface temperatures were maintained at 200 °C and 250 °C, respectively. Compounds were tentatively identified by comparison of the obtained mass spectra with those in the National Institute of Standards and Technology mass spectral library.

**1.3. IFT measurement**

The IFT between TO and aqueous surfactant solutions was measured using a drop shape analyzer (DSA100, KRÜSS, Germany) with the pendant drop method. TO was used as the surrounding oil phase, and droplets of the aqueous phase containing BS, LEC, SAP, or their combinations were formed at the tip of a syringe needle (NE44 or NE81, KRÜSS, Germany). The droplet profiles were recorded using an integrated video camera, and the IFT values were calculated by fitting the droplet shape to the Young-Laplace equation using the instrument software. All measurements were performed at 25 °C.

**1.4. Bio-TEM**

Bacterial cells were fixed using a primary fixation solution containing 2% glutaraldehyde and 2% formaldehyde prepared in 0.1 M sodium cacodylate buffer (pH 7.2) at 4 °C for 4–20 h. Following fixation, the samples were washed three times with 0.05 M sodium cacodylate buffer (pH 7.2) for 10 min each, with centrifugation at 16,100 × g for 20 s between washing steps. A secondary fixation was then carried out using 1% osmium tetroxide in 0.1 M sodium cacodylate buffer (pH 7.2) at 4 °C for 75–90 min, followed by centrifugation under the same conditions. The fixed cells were subsequently rinsed three times with distilled water and subjected to dehydration through a graded ethanol series (30%, 50%, 70%, 80%, 90%, and three changes of 100%), with each step performed for 35–40 min and followed by centrifugation. The dehydrated samples were transitioned into resin using 100% propylene oxide twice, followed by infiltration with mixtures of Embed 812 resin and propylene oxide at different ratios (2:1, 1:1, and 1:2). Final infiltration was performed with pure resin under vacuum for approximately 24 h. The embedded samples were polymerized at 65 °C for 12–18 h and allowed to cool to room temperature. Ultrathin sections (~100 nm) were obtained using an ultramicrotome and mounted onto 200-mesh copper grids. The sections were stained with uranyl acetate followed by lead citrate prior to observation. TEM imaging was conducted using a Talos L120C (Thermo Fisher Scientific, USA) operated at an accelerating voltage of 80 kV.

**2. Supplementary table**

**Table S1.** Calorimetric estimation of ultrasonication parameters under the applied processing conditions.

| **Parameter** | **Value** |
| --- | --- |
| Estimated acoustic power (W) | 2.1 |
| Actual sonication time (s) | 300 |
| Total energy input (J) | 633 |
| Energy density (J/mL) | 63.3 |

Acoustic power (*P*) was calculated using *P* = m*C*_p_*ΔT*/*t*, where *m* is the mass of water, *C*_p_ is the specific heat capacity of water, *ΔT* is the temperature increase, and *t* is the sonication time.

**3. Supplementary figures**


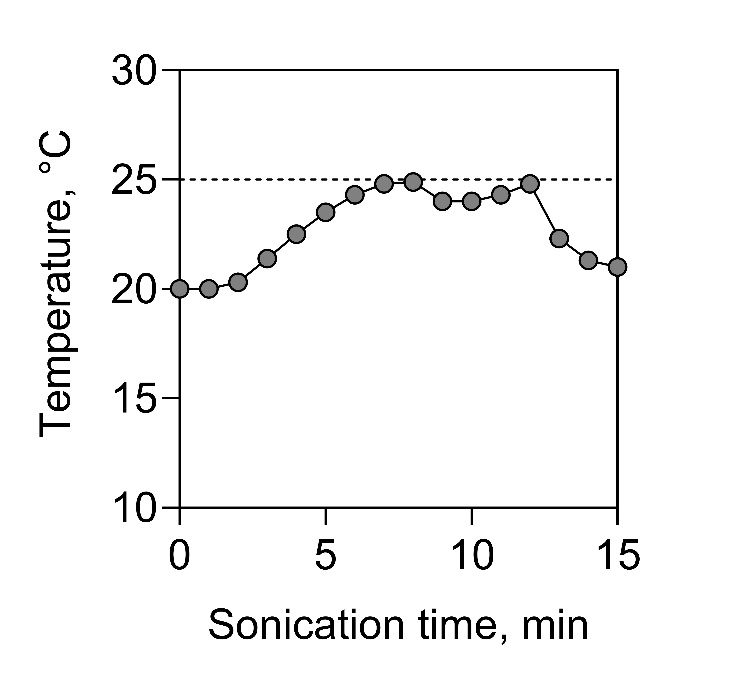


**Fig. S1.** Temperature profile of the sample during ultrasonication under pulse-mode and ice-bath conditions.


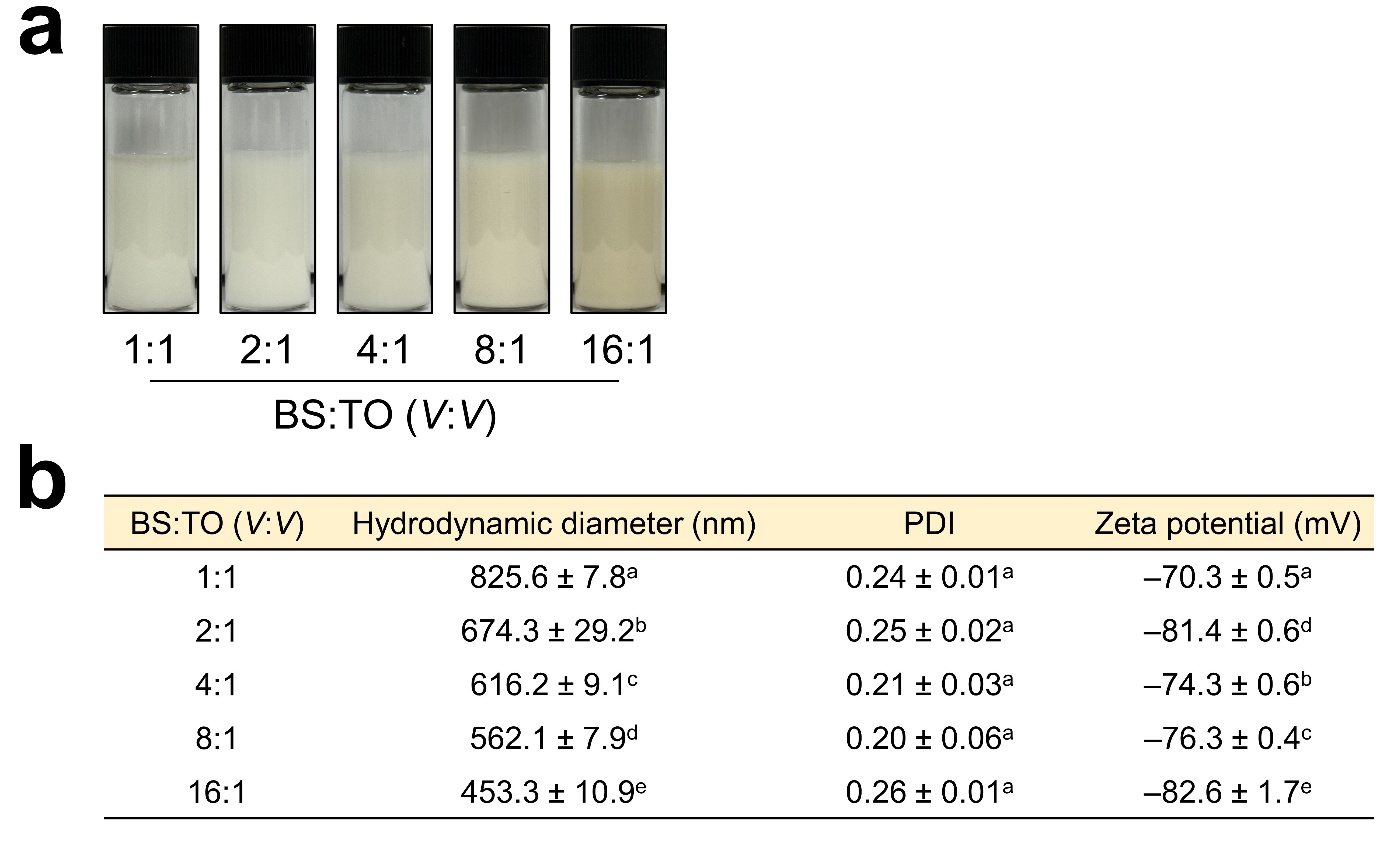


**Fig. S2.** **Effect of BS-to-TO ratio on the formation and droplet characteristics of BS-based NEs.** **(a)** Visual appearance of NEs prepared with different BS-to-TO volume ratios (BS:TO, *V*:*V*). **(b)** Hydrodynamic diameter, PDI, and zeta potential of NEs formulated at different BS:TO ratios, as determined by DLS. Values are expressed as mean ± standard deviation (*n* = 3). Different superscript letters within the same column indicate statistically significant differences (*p* < 0.05), as determined by ANOVA.


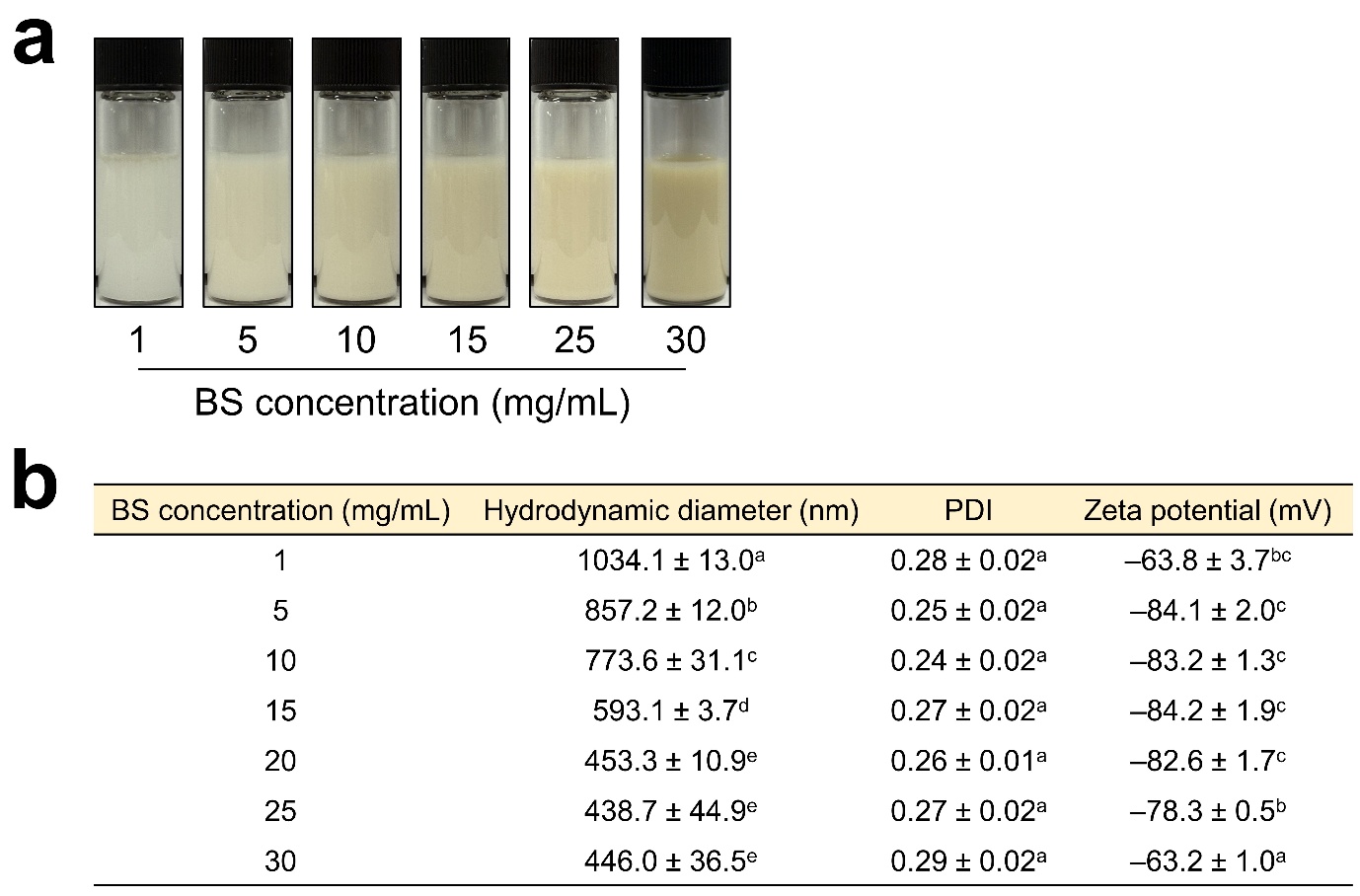


**Fig. S3.** **Effect of BS concentration on the formation and droplet characteristics of BS-based NEs.** **(a)** Visual appearance of NEs prepared with different concentrations of BS. **(b)** Hydrodynamic diameter, PDI, and zeta potential of NEs formulated at different BS concentrations, as determined by DLS. Values are expressed as mean ± standard deviation (*n* = 3). Statistical significance was determined by ANOVA, and different superscript letters within the same column indicate significant differences (*p* < 0.05).


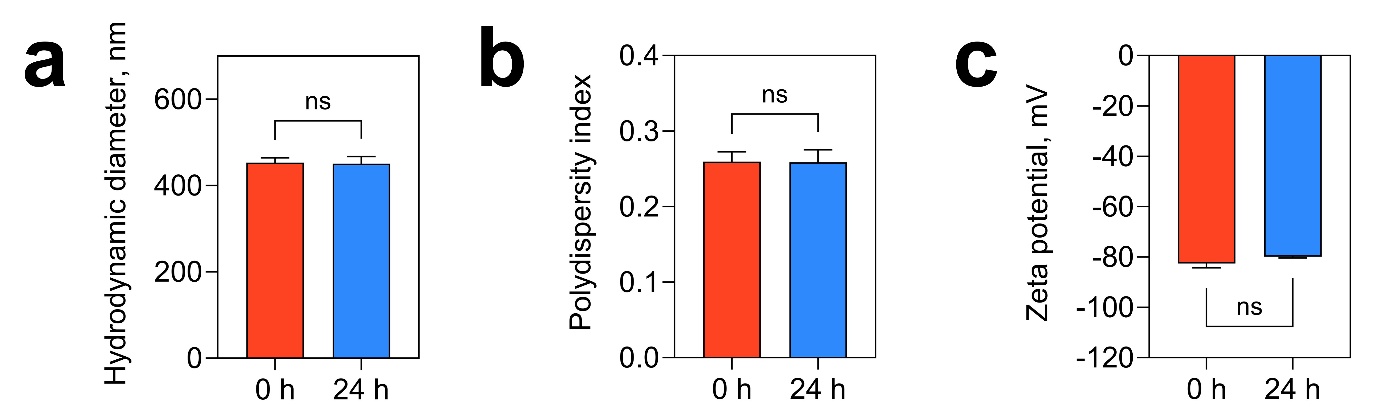


**Fig. S4. Short-term physicochemical stability of the selected BS-NE formulation during 24 h storage.** **(a)** Size. **(b)** PDI. **(c)** Zeta potential.


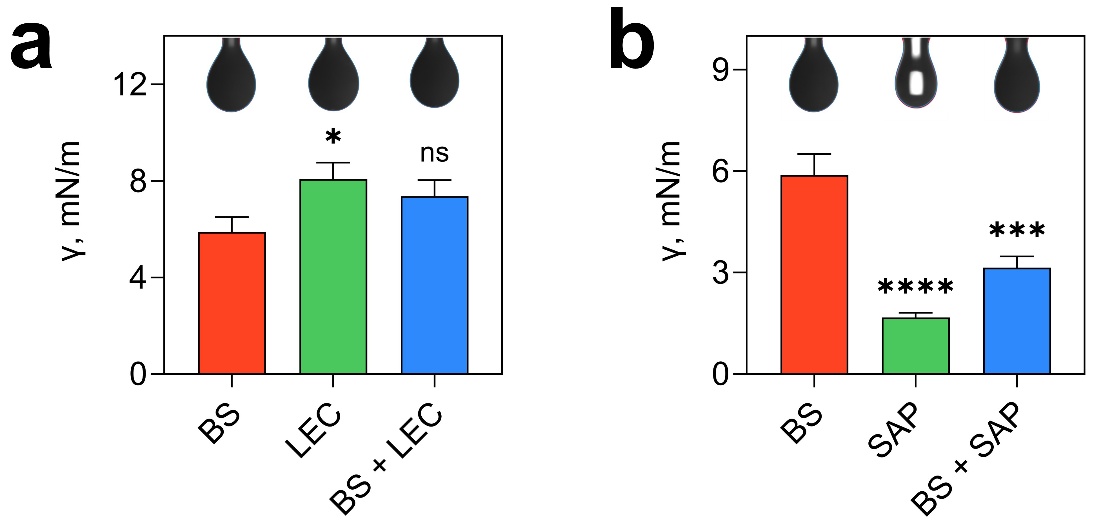


**Fig. S5.** **Interfacial tension between TO and aqueous solutions of individual and combined natural surfactants.** **(a)** Interfacial tension of BS, LEC, and BS-LEC systems (*n* = 5). **(b)** Interfacial tension of BS, SAP, and BS-SAP systems (*n* = 5). **p* < 0.05, ****p* < 0.001, and *****p* < 0.0001 indicate significant differences compared with BS, while ns denotes no statistically significant difference.


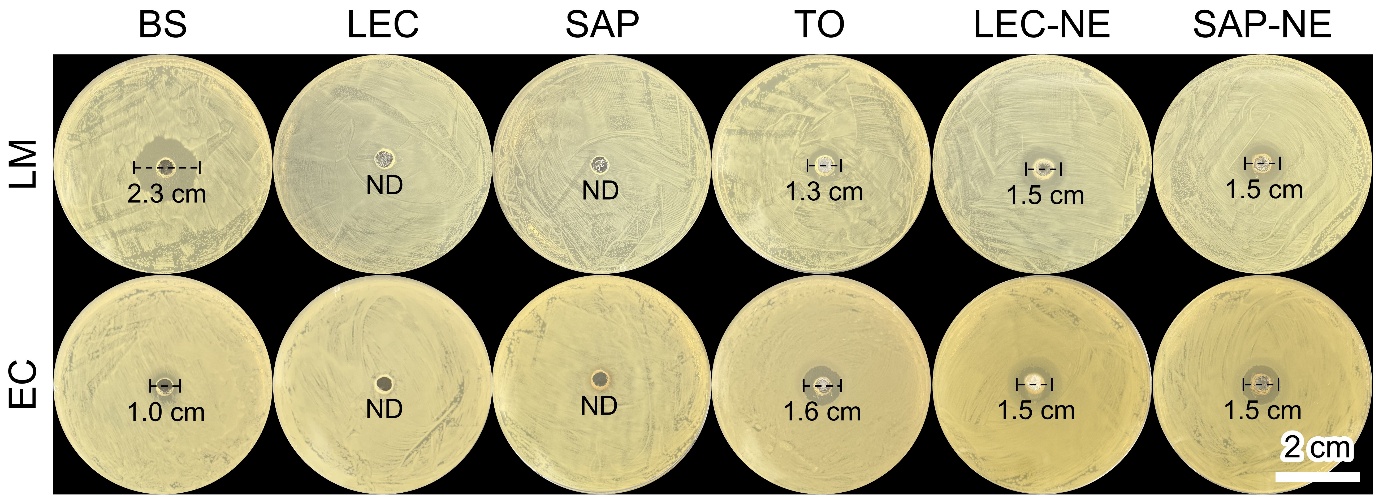


**Fig. S6.** **Agar diffusion assay showing inhibition zones against *Listeria monocytogenes* (LM) and *Escherichia coli* O157 (EC) for individual components and natural surfactant (LEC and SAP)-based NE formulations.** BS, LEC, SAP, and TO were tested at concentrations equivalent to those used in the NE formulations. ND denotes no detectable zone of inhibition.
